# Quiescent stem cell marker genes in glioma gene networks are sufficient to distinguish between normal and glioblastoma (GBM) samples

**DOI:** 10.1038/s41598-020-67753-5

**Published:** 2020-07-02

**Authors:** Shradha Mukherjee

**Affiliations:** Unaffiliated, Los Angeles, CA 90017 USA

**Keywords:** Cancer, Computational biology and bioinformatics, Stem cells

## Abstract

Grade 4 glioma or GBM has poor prognosis and is the most aggressive grade of glioma. Accurate diagnosis and classification of tumor grade is a critical determinant for development of treatment pathway. Extensive genomic sequencing of gliomas, different cell types, brain tissue regions and advances in bioinformatics algorithms, have presented an opportunity to identify molecular markers that can complement existing histology and imaging methods used to diagnose and classify gliomas. ‘Cancer stem cell theory’ purports that a minor population of stem cells among the heterogeneous population of different cell types in the tumor, drive tumor growth and resistance to therapies. However, characterization of stem cell states in GBM and ability of stem cell state signature genes to serve as diagnostic or prognostic molecular markers are unknown. In this work, two different network construction algorithms, Weighted correlation network analysis (WGCNA) and Multiscale Clustering of Geometric Network (MEGENA), were applied on publicly available glioma, control brain and stem cell gene expression RNA-seq datasets, to identify gene network regulatory modules associated with GBM. Both gene network algorithms identified consensus or equivalent modules, HuAgeGBsplit_18 (WGCNA) and c1_HuAgeGBsplit_32/193 (MEGENA), significantly associated with GBM. Characterization of HuAgeGBsplit_18 (WGCNA) and c1_HuAgeGBsplit_32/193 (MEGENA) modules showed significant enrichment of rodent quiescent stem cell marker genes (GSE70696_QNPbyTAP). A logistic regression model built with eight of these quiescent stem cell marker genes (GSE70696_QNPbyTAP) was sufficient to distinguish between control and GBM samples. This study demonstrates that GBM associated gene regulatory modules are characterized by diagnostic quiescent stem cell marker genes, which may potentially be used clinically as diagnostic markers and therapeutic targets in GBM.

## Introduction

GBM is a tumor that occurs in brain and can spread to spinal cord^1^. Prognosis of GBM is poor and treatment options are limited, with most patients not surviving the disease^2^. For diagnosis and treatment of GBM, identification of glioma grade is key to devise tumor specific treatment pathways^3^. Gliomas are classified as grade 2, grade 3 and grade 4 gliomas, with increasing aggressiveness and decreasing survival rates^4,5^. Gliomas of different grades do not respond uniformly to treatment as they are distinctly different from each other^6^. To properly test efficacy of drugs and biologics in glioma clinical trials, it is important to understand how their effectiveness changes in tumor grades^7^. Research and market for GBM treatment is predicted to rapidly grow from $465 million in 2016 to $1 billion by 2025^8^.

Glioma grade is a key determinant of metastasis and tumor relapse in patients. Presently, tumor imaging, limited molecular profiling and histological analysis of tumor biopsy are techniques used to grade gliomas in clinical settings^7^. However, application of a wider range of molecular analysis can complement these existing techniques and improve glioma classification. Cancer therapy driven by molecular classification holds promise to improve patient specific tumor grading for application of precision medicine for improved treatment outcome. It is speculated that gliomas originate from mutation in proliferative stem cells in the brain and number of mutations grow during the course of tumor development. Therefore, it maybe possible to grade gliomas at the molecular level based on the stem cell signature genes^9,10^. Moreover, stem cell signature genes could potentially serve as targets for gene therapy and drugs^11,12^.

‘Stem cell theory of cancer’ states that resistance of cancer to chemotherapy and radiotherapy, is due to resident tumor stem cells^13^. Chemotherapy and radiotherapy treatments work by specifically targeting proliferative stem cells, but non-proliferative stem cells that are in a resting state called quiescence, escape this treatment strategy^14^. Resting stem cells in cancer under suitable conditions can become proliferative, replenish tumor cells and mutate further to reestablish the tumor after chemotherapy and radiotherapy. Thus, it is essential to determine stem cell molecular markers underlying GBM associated gene networks to improve treatment outcome and patient survival.

An emerging field of drug development is ‘network medicine’, where a combination of drugs are picked to target multiple subnetworks or modules of a disease gene regulatory network^15,16^. ‘Omics’ or genomics data and biomedical literature text data are two major sources of gene and gene interaction information in human health and disease. Both omics data and text data resources are used to identify druggable gene networks and pathways underlying disease symptoms. Extensive research endeavors led to establish collections of RNA-sequencing (RNA-seq), chromatin immunoprecipitation sequencing (ChIP-seq), ES (Exome Sequencing), Whole Exome Sequencing (WES), high-throughput proteomics and other ‘omics’ patient data^17^. The Cancer Genome Atlas (TCGA), International Cancer Genome Consortium (ICGC), Chinese Glioma Genome Atlas (CGGA) and NCBI GEO are some examples of publicly available databases for collection and storage of patient omics data^18–21^. Additionally, NCBI GEO archives a multitude of genome-wide molecular transcriptome profiles, including stem cell transcriptome data from human and non-human animal species^19^. Presently, a RNA-seq meta-analysis was performed to identify stem cell enriched GBM modules and develop a diagnostic model based on stem cell genes, which is able to distinguish between control and GBM samples.

Here, lists of quiescent and proliferative state stem cell signature genes were identified underlying GBM specific gene regulatory modules. Two network analysis methods, WGCNA and MEGENA, were utilized to build a glioma gene regulatory network and GBM associated sub-networks or modules based on gene–gene co-expression patterns^22–24^. Both methods identified comparable GBM associated modules, which suggests that GBM modules identified in this study are biologically robust. GBM modules, HuAgeGBsplit_18 (WGCNA) and its equivalent c1_HuAgeGBsplit_32/193 (MEGENA), were enriched with quiescent stem cell signature genes (GSE70696_QNPbyTAP) and expression of these quiescent stem cell signature genes was sufficient to build a logistic regression diagnostic model capable of distinguishing between control and GBM samples. In summary, a set of quiescent stem cell markers enriched in GBM modules was identified with potential application in diagnostic GBM screening.

## Methods

### Glioma RNA-seq data sources

RNA-seq gene expression datasets were obtained from NCBI Sequence Read Archive (SRA) and NCBI Gene Expression Omnibus (GEO)^19,25^. RNA-seq gene expression datasets SRP027383 and SRP091303 for glioma belonging to grade 2, grade 3 and GBM (grade 4) was obtained from NCBI SRA^18^. SRP027383 dataset is also available on NCBI GEO under series number GSE48865. RNA-seq gene expression datasets were obtained from NCBI GEO, belonging to various parts of brain to use as controls: cortex (series numbers GSE64810, GSE53697), hippocampus (series number GSE67333) and optic chiasma (series number GSE100297)^26–29^.

### Stem cell RNA-seq data sources

Five different proliferative and quiescent stem cell datasets were used in this study. Two human RNA-seq gene expression datasets were obtained from NCBI GEO for GBM grown in quiescent and proliferative culture conditions, with series numbers GSE93991 and GSE114574^30,31^. Both RNA-seq and microarray gene expression datasets for adult rodent brain stem cells in proliferative and quiescent states, were obtained from NCBI GEO with series numbers GSE68270, GSE70696 and GSE99777^32–34^.

### Processing of RNA-seq reads

RNA-seq reads were mapped to genome and genes were annotated as previously described^33,35^. Briefly, sra raw read files were converted to fastq using sratoolkit.2.9.1 and quality was assessed with fastqc_v0.11.7^25,36^. Alignment of RNA-seq raw reads on reference genome was done with tophat 2.1.1 aligner using gene annotation information with parameters: tophat2-b2 -very-fast -no-coverage-search -no-novel-juncs^37^. Following reference genome and annotation files were used: (1) for human: Homo_sapiens.GRCh38.dna.primary_assembly.fa and Homo_sapiens.GRCh38.92.gtf, (2) for mouse: Mus_musculus.GRCm38.dna.primary_assembly.fa and Mus_musculus.GRCm38.92.gtf, (3) for rat: rnor6_ensemble_seq_whole_genome.fa and Rattus_norvegicus.Rnor_6.0.95.gtf. Mapped output bam files were analyzed with HTSeq 0.10.0 to estimate gene abundance and obtain count reads overlapping a gene with parameters: htseq-count -r pos -t gene_name^38^.

### Glioma gene network analysis with WGCNA and MEGENA

Gene counts from HTSeq 0.10.0 were normalized to TPM values and filtered to keep values > 1 in atleast 2 samples^39^. TPM values were further scaled to log2TPM + 1 and visualized with volcano plots, barplots and density plots in ggplot2_3.1.0 R package^40^. To compare samples before and after TPM normalization, prcomp function in stats4_3.5.0 R package and corrplot_0.84 R package were used to perform PCA and correlation analysis, respectively^40,41^. To remove effects of covariants other than glioma, such as gender, study or batch, age, tissue and surrogate variables (hidden variables) on gene expression, a surrogate variable adjustment and linear model (SVA + LM) method was applied to log2TPM + 1 values, following previously published methods using sva_3.30.1 and limma_3.38.3 R packages^35,42–45^. After SVA + LM adjustment the resultant normalized log2TPM + 1 gene expression was used to build a glioma gene network with subnetworks or modules using WGCNA_1.66 and MEGENA_1.3.7 R packages^23,24,35,46^.

In WGCNA, default parameters and a minimum module size of 100 was used to calculate a topology overlap matrix (TOM) based on gene expression correlations. Hierarchical clustering was then used to build a glioma gene network consisting of interconnected subnetworks or modules^22,23^. In MEGENA, default parameters and a minimum module size of 100 was used to calculate a planar filtered network (PFN) from gene expression correlations. Multiscale clustering method was applied to build a glioma gene network consisting of interconnected subnetworks or modules^24^. WGCNA computes scale-free or single scale networks, while MEGENA computes multi-scale networks to include different possible variations of gene–gene interactions. Therefore, in MEGENA a given gene or node can be part of multiple modules representing different possible interactions, while in WGCNA a given gene or node is assigned to only a single module. To determine module trait correlations, module eigengenes were computed with moduleEigengenes R function and correlations were calculated^22^. To compare WGCNA and MEGENA modules, previously published module preservation analysis and hypergeometric enrichment tests were used^35,47^. Briefly, hypergeometric test was implemented with userListEnichment R function widely used to compare WGCNA gene network modules with each other and with user supplied gene lists^22,23,46^. Both module preservation and userListEnrichment R functions are from WGCNA^23,47^. After module preservation analysis between WGCNA and MEGENA modules, significant overlap of genes between WGCNA and MEGENA modules was done with userListEnrichment R function for all WGCNA and MEGENA modules i.e. each WGCNA module was compared with each MEGENA module. WGCNA and MEGENA modules that significantly overlapped and were significantly associated with GBM were retained.

### Stem cell differential gene expression analysis with limma, edgeR and simple comparison of means

Differentially expressed genes (DEGs) between proliferative and quiescent stem cell states were identified using R packages limma_3.38.3, edgeR_3.24.3 and simple comparison expression means^18,45,48^. To calculate genes enriched in proliferative stem cells, gene expression of samples annotated to proliferative stem cells were compared with gene expression of samples annotated to quiescent stem cells for each of the five datasets (GSE68270, GSE70696, GSE99777, GSE93991 and GSE114574). Similarly, to calculate genes enriched in quiescent stem cells, gene expression of samples annotated to quiescent stem cells were compared with gene expression of samples annotated to proliferative stem cells for each of the five datasets (GSE68270, GSE70696, GSE99777, GSE93991 and GSE114574). In simple comparison of means method, mean expression of genes were simply compared between proliferative and quiescent stem cell states to determine DEGs. In limma and edgeR model design included variables stem cell state, study or batch, gender, age and tissue, and stem cell states were contrasted to determine DEGs, while all other variables were held constant in the model. In limma and edgeR methods Benjamini and Hochberf (BH) corrections for multiple testing is included as a large number of genes were included in analysis^49^. DEGs with BH corrected adjP-values < 0.05 and fold change > 1.25 were considered significant DEGs. To visualize gene expression values of DEGs volcano plots, barplots and density plots were made using ggplot2_3.1.1 R package^40^. Consensus DEGs were obtained by overlapping DEG lists produced by limma, edgeR and simple comparison of means with a significance of overlap p-value < 0.05 as calculated with GeneOverlap_1.18.0 and visualized by VennDiagram_1.6.20 R packages^50,51^. Consensus DEGs that belonged to atleast two of the three DEG lists produced by limma, edgeR and simple comparison of means were designated significantly enriched genes or DEGs in proliferative and quiescent stem cell states—simply referred to as (1) proliferative stem cell marker genes and (2) quiescent stem cell marker genes.

Following is a detailed description of all DEG analysis contrasts, sample size for each dataset and abbreviations used to represent proliferative and quiescent stem cell marker genes: (A) Adult proliferative neural progenitor cells (PNPCs) vs adult quiescent neural stem cells (QNSCs) DEG analysis to identify genes enriched in PNPCs relative to QNSCs in mouse dataset with series number GSE68270 and sample size of 4 each, abbreviated as GSE68270_PNPCbyQNSC^32^ (B) Adult quiescent neural stem cells (QNSCs) vs adult proliferative neural progenitor cells (PNPCs) DEG analysis to identify genes enriched in QNSCs relative to PNPCs in mouse dataset with series number GSE68270 and sample size of 4 each, abbreviated as GSE68270_QNSCbyPNPC^32^ (C) Adult hippocampal stem cells in proliferative condition or transient amplifying progenitor cells (TAPs) vs adult hippocampal stem cells in quiescent condition or quiescent progenitor cells (QNPs) DEG analysis to identify genes enriched in TAPs relative to QNPs in rat dataset with series number GSE70696 and sample size of 2 each, abbreviated as GSE70696_TAPbyQNP^33^ (D) Adult hippocampal stem cells in quiescent condition or quiescent progenitor cells (QNPs) vs adult hippocampal stem cells in proliferative condition or transient amplifying progenitor cells (TAPs) DEG analysis to identify genes enriched in QNPs relative to TAPs in rat dataset with series number GSE70696 and sample size of 2 each, abbreviated as GSE70696_QNPbyTAP^33^ (E) Adult proliferative sub-ventricular zone stem cells (PSVZSCs) vs adult quiescent sub-ventricular zone stem cells (QSVZSCs) DEG analysis to identify genes enriched in PSVZSCs relative to QSVZSCs in mouse microarray dataset with series number GSE99777 and sample size of 3 each, abbreviated as GSE99777_PSVZSCbyQSVZSC^34^ (F) Adult quiescent sub-ventricular zone stem cells (QSVZSCs) vs adult proliferative sub-ventricular zone stem cells (PSVZSCs) DEG analysis to identify genes enriched in QSVZSCs relative to PSVZSCs in mouse microarray dataset with series number GSE99777 and sample size of 3 each, abbreviated as GSE99777_QSVZSCbyPSVZSC^34^ (G) GBM cells cultured in proliferative condition or proliferative GBM cells (PGBCs) vs GBM cells cultured in quiescent condition or quiescent GBM cells (QGBCs) DEG analysis to identify genes enriched in PGBCs relative to QGBCs in human dataset with series number GSE93991 and sample size of 9 and 6, respectively, abbreviated as GSE93991_PGBCbyQGBC^30^ (H) GBM cells cultured in quiescent condition or quiescent GBM cells (QGBCs) vs GBM cells cultured in proliferative condition or proliferative GBM cells (PGBCs) DEG analysis to identify genes enriched in QGBCs relative to PGBCs in human dataset with series number GSE93991 and sample size of 6 and 9, respectively, abbreviated as GSE93991_QGBCbyPGBC^30^ (I) GBM organoids cultured in proliferative condition or proliferative GBM organoids (PGBOs) vs GBM organoids cultured in quiescent condition or quiescent GBM cells (QGBOs) DEG analysis to identify genes enriched in PGBOs relative to QGBOs in human dataset with series number GSE114574 and sample size 6, abbreviated as GSE114574_PGBObyQGBO^31^ and (J) GBM organoids cultured in quiescent condition or quiescent GBM organoids (QGBOs) vs GBM organoids cultured in proliferative condition or proliferative GBM organoids (PGBOs) DEG analysis to identify genes enriched in QGBOs relative to PGBOs in human dataset with series number GSE93991 and sample size of 6, abbreviated as GSE114574_QGBObyPGBO^31^.

### Enrichment of proliferative and quiescent stem cell marker genes in glioma modules

Enrichment of proliferative and quiescent stem cell marker genes identified by differential gene expression analysis above, in WGCNA and MEGENA modules was determined using useListEnrichment R function^22,23,46^. Additionally, a supplementary table containing a set of 336 genes potentially involved in transition of GBM from stem-like state to differentiation identified by SWIM tool were downloaded directly from the published paper^52,53^. Enrichment of SWIM GBM list in WGCNA and MEGENA modules was also determined using useListEnrichment R function^22,23,46^. Briefly, hypergeometric test was implemented with userListEnichment R function from WGCNA R package^23,46^. Hypergeometric test was implemented with userListEnrichment R function to determine: (A) Significant overlap of a WGCNA module set vs a set of proliferative stem cell marker genes. This was done for all WGCNA modules and all proliferative stem cell marker gene sets (GSE68270_PNPCbyQNSC, GSE70696_TAPbyQNP, GSE99777_PSVZSCbyQSVZSC, GSE93991_PGBCbyQGBC and GSE114574_PGBObyQGBO). (B) Significant overlap of a WGCNA module set vs a set of quiescent stem cell marker genes. This was done for all WGCNA modules and all quiescent stem cell marker gene sets (GSE68270_QNSCbyPNPC, GSE70696_QNPbyTAP, GSE99777_QSVZSCbyPSVZSC, GSE93991_QGBCbyPGBC and GSE114574_QGBObyPGBO). (C) Significant overlap of a WGCNA module set vs a set of SWIM 336 GBM gene list. This was done for all WGCNA modules and the SWIM 336 GBM gene list. (D) Significant overlap of a MEGENA module set vs a set of proliferative stem cell marker genes. This was done for all WGCNA modules and all proliferative stem cell marker gene sets (GSE68270_PNPCbyQNSC, GSE70696_TAPbyQNP, GSE99777_PSVZSCbyQSVZSC, GSE93991_PGBCbyQGBC and GSE114574_PGBObyQGBO). (E) Significant overlap of a MEGENA module set vs a set of quiescent stem cell marker genes. This was done for all WGCNA modules and all quiescent stem cell marker gene sets (GSE68270_QNSCbyPNPC, GSE70696_QNPbyTAP, GSE99777_QSVZSCbyPSVZSC, GSE93991_QGBCbyPGBC and GSE114574_QGBObyPGBO). And (F) Significant overlap of a MEGENA module set vs a set of SWIM 336 GBM gene list. This was done for all MEGENA modules and the SWIM 336 GBM gene list. The results from (A) to (F) analysis were filtered to retain WGCNA and MEGENA GBM modules significantly enriched with proliferative and quiescent stem cell marker genes.

### Gene ontology (GO) analysis of GBM modules significantly enriched with proliferative and quiescent stem cell marker genes

EnrichR_1.0 R package was used to determine biological process gene ontology (GO) functional characterization of the Glioblastoma associated module gene members and p-value < 0.05 was kept as significant^54^.

### GBM logistic regression diagnostic model with stem cell genes and Hosmer–Lemeshow goodness of fit (GOF) test

To distinguish between control and GBM samples, a logistic regression model was built with stem cell marker genes most significantly enriched in GBM modules and most upregulated in stem cell DEG list. Logistic regression was performed with glm R function from stats4_3.5.0 R package with selected genes and gene–gene interaction terms: glm(formula = DiseaseGrade_4 ~ gene1 + gene2 + …gene1*gene2*…, family = binomial(link = logit), data = data, na.action = na.exclude). Hosmer–Lemeshow GOF test was used to evaluate how well logistic regression model fits data using R package ResourceSelection v0.3–5^55,56^. In other words, Hosmer–Lemeshow GOF test was used to evaluate how well probability values predicted by logistic regression model (expected probabilities) matched observed probabilities. For Hosmer–Lemeshow GOF test, H0 (null hypothesis) is that observed and expected probabilities do not differ significantly (good fit), while Ha (alternate hypothesis) is that observed and expected probabilities differ significantly (poor fit). A p-value < 0.05 and a large difference between observed and expected probabilities, indicates poor model fit on data so model can be rejected (reject Ho, accept Ha). On the other hand, p-value > 0.05 and a small difference between observed and expected probabilities, indicates there is no evidence of poor fit so model can be accepted as a good fit (accept Ho).

### Consensus significantly enriched genes or DEGs in stem cell proliferation and quiescence

To determine proliferative and quiescent stem cell marker genes common between all five stem cell datasets (GSE68270, GSE70696, GSE99777, GSE93991 and GSE114574), the following consensus significantly enriched genes or DEGs in proliferative and quiescent states were overlapped, respectively, using online tool https://www.molbiotools.com/listcompare.html : (A) For proliferation comparison of (GSE68270_PNPCbyQNSC + GSE70696_TAPbyQNP + GSE99777_PSVZSCbyQSVZSC + GSE93991_PGBCbyQGBC + GSE114574_PGBObyQGBO) and (B) For quiescence comparison of (GSE68270_QNSCbyPNPC + GSE70696_QNPbyTAP + GSE99777_QSVZSCbyPSVZSC + GSE93991_QGBCbyPGBC + GSE114574_QGBObyPGBO).

To determine proliferative and quiescent stem cell marker genes common to both normal stem cells and GBM in culture, following consensus significantly enriched genes or DEG lists in proliferative and quiescent states were overlapped, respectively, using online tool https://www.molbiotools.com/listcompare.html (A) comparison of normal stem cells (GSE68270_PNPCbyQNSC + GSE70696_TAPbyQNP + GSE99777_PSVZSCbyQSVZSC) and GBM cell cultures (GSE93991_PGBCbyQGBC + GSE114574_PGBObyQGBO) DEG lists in proliferative conditions (B) Comparison of normal stem cells (GSE68270_QNSCbyPNPC + GSE70696_QNPbyTAP + GSE99777_QSVZSCbyPSVZSC) and GBM cell cultures (GSE93991_QGBCbyPGBC + GSE114574_QGBObyPGBO) DEGs in quiescent conditions.

### Code availability

The computational pipeline used in this work is open-sourced and available both on github https://github.com/smukher2/GithubScientificReportsGlioblastomaStemApril2020 and protocol exchange research square https://dx.doi.org/10.21203/rs.3.pex-977/v1.

## Results

### SVA + LM approach reduces batch effects in RNA-seq datasets

**Figure 1:**
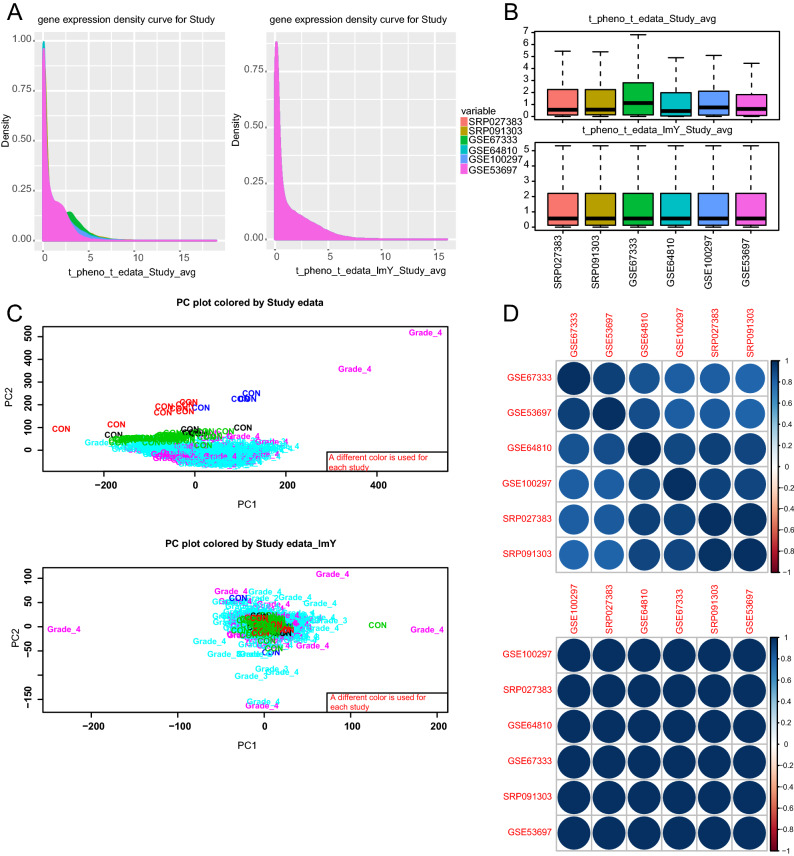
Effect of SVA + LM adjustment on Glioblastoma (SRP027383, SRP091303) and control brain (GSE67333, GSE64810, GSE100297, GSE53697) RNA-seq studies. (**A**) Density plot representation of gene expression per study before and after SVA + LM adjustment (**B**) Box and whisker plot representation of gene expression per study before and after SVA + LM adjustment. (**C**) Principal component (PC) projections of datasets before and after SVA + LM adjustment. (**D**) Pearson correlation plot of the studies before and after SVA + LM adjustment.

To identify GBM specific transcriptome features, a meta-analysis was performed with glioma and control brain human RNA-seq samples. SVA + LM normalization reduced variability due to batch effects as indicated by greater overlap of expression data in density plots after SVA + LM normalization (Fig. 1A). Box-Whiskers plots showed a slightly skewed mean expression in GSE67333 dataset that was fixed by SVA + LM normalization (Fig. 1B). PCA plots showed that SVA + LM normalization reduces dispersion of samples from same study (Fig. 1C). Correlation plots to evaluate correlation of gene expression values among different studies, showed an increase in positive correlation after SVA + LM normalization (Fig. 1D). Thus, SVA + LM normalization may be used to achieve reduction in batch effects in global gene expression when RNA-seq studies are combined.

### Network analysis reveals GBM associated modules within glioma network

Glioma gene co-expression networks were constructed with WGCNA and MEGENA to uncover underlying molecular mechanisms. WGCNA identified 39 modules with largest module HuAgeGBsplit_01 comprising of 7,969 genes and smallest module HuAgeGBsplit_38 comprising of 113 genes (Table 1). MEGENA identified 235 modules with largest module c1_HuAgeGBsplit_13 comprising of 1,483 genes and smallest modules c1_HuAgeGBsplit_1081/181/1895 comprising of 100 genes each (Table 2).

**Table 1:** Number of genes in each WGCNA module.

	WGCNA_Module_Names	Number_of_Genes		WGCNA_Module_Names	Number_of_Genes
1	HuAgeGBsplit_01	7,969	21	HuAgeGBsplit_21	365
2	HuAgeGBsplit_02	7,732	22	HuAgeGBsplit_22	345
3	HuAgeGBsplit_03	1,234	23	HuAgeGBsplit_23	321
4	HuAgeGBsplit_04	938	24	HuAgeGBsplit_24	312
5	HuAgeGBsplit_05	809	25	HuAgeGBsplit_25	305
6	HuAgeGBsplit_06	727	26	HuAgeGBsplit_26	303
7	HuAgeGBsplit_07	724	27	HuAgeGBsplit_27	249
8	HuAgeGBsplit_08	673	28	HuAgeGBsplit_28	246
9	HuAgeGBsplit_09	643	29	HuAgeGBsplit_29	242
10	HuAgeGBsplit_10	629	30	HuAgeGBsplit_30	234
11	HuAgeGBsplit_11	581	31	HuAgeGBsplit_31	219
12	HuAgeGBsplit_12	496	32	HuAgeGBsplit_32	204
13	HuAgeGBsplit_13	493	33	HuAgeGBsplit_33	199
14	HuAgeGBsplit_14	492	34	HuAgeGBsplit_34	181
15	HuAgeGBsplit_15	488	35	HuAgeGBsplit_35	161
16	HuAgeGBsplit_16	487	36	HuAgeGBsplit_36	121
17	HuAgeGBsplit_17	421	37	HuAgeGBsplit_37	121
18	HuAgeGBsplit_18	394	38	HuAgeGBsplit_38	113
19	HuAgeGBsplit_19	394	39	HuAgeGBsplit_00	18
20	HuAgeGBsplit_20	381

**Table 2:** Number of genes in each MEGENA module.

	MEGENA_Module_Names	Number_of_Genes		MEGENA_Module_Names	Number_of_Genes		MEGENA_Module_Names	Number_of_Genes
1	c1_HuAgeGBsplit_13	1,483	81	c1_HuAgeGBsplit_76	256	161	c1_HuAgeGBsplit_331	143
2	c1_HuAgeGBsplit_26	1,409	82	c1_HuAgeGBsplit_1421	255	162	c1_HuAgeGBsplit_293	141
3	c1_HuAgeGBsplit_10	1,407	83	c1_HuAgeGBsplit_1905	254	163	c1_HuAgeGBsplit_681	141
4	c1_HuAgeGBsplit_6	1,385	84	c1_HuAgeGBsplit_90	242	164	c1_HuAgeGBsplit_1417	140
5	c1_HuAgeGBsplit_180	1,182	85	c1_HuAgeGBsplit_48	241	165	c1_HuAgeGBsplit_212	140
6	c1_HuAgeGBsplit_87	1,144	86	c1_HuAgeGBsplit_1364	234	166	c1_HuAgeGBsplit_84	140
7	c1_HuAgeGBsplit_11	1,070	87	c1_HuAgeGBsplit_294	228	167	c1_HuAgeGBsplit_367	138
8	c1_HuAgeGBsplit_450	969	88	c1_HuAgeGBsplit_58	228	168	c1_HuAgeGBsplit_161	137
9	c1_HuAgeGBsplit_83	930	89	c1_HuAgeGBsplit_179	227	169	c1_HuAgeGBsplit_1195	136
10	c1_HuAgeGBsplit_15	907	90	c1_HuAgeGBsplit_59	224	170	c1_HuAgeGBsplit_1590	136
11	c1_HuAgeGBsplit_22	899	91	c1_HuAgeGBsplit_191	223	171	c1_HuAgeGBsplit_1359	134
12	c1_HuAgeGBsplit_21	875	92	c1_HuAgeGBsplit_27	223	172	c1_HuAgeGBsplit_287	134
13	c1_HuAgeGBsplit_9	866	93	c1_HuAgeGBsplit_80	223	173	c1_HuAgeGBsplit_193	133
14	c1_HuAgeGBsplit_443	838	94	c1_HuAgeGBsplit_305	221	174	c1_HuAgeGBsplit_704	133
15	c1_HuAgeGBsplit_65	830	95	c1_HuAgeGBsplit_306	221	175	c1_HuAgeGBsplit_1245	132
16	c1_HuAgeGBsplit_5	804	96	c1_HuAgeGBsplit_329	218	176	c1_HuAgeGBsplit_1955	132
17	c1_HuAgeGBsplit_16	799	97	c1_HuAgeGBsplit_1398	217	177	c1_HuAgeGBsplit_597	131
18	c1_HuAgeGBsplit_20	719	98	c1_HuAgeGBsplit_175	217	178	c1_HuAgeGBsplit_583	130
19	c1_HuAgeGBsplit_24	708	99	c1_HuAgeGBsplit_55	215	179	c1_HuAgeGBsplit_1297	129
20	c1_HuAgeGBsplit_25	704	100	c1_HuAgeGBsplit_185	208	180	c1_HuAgeGBsplit_1954	129
21	c1_HuAgeGBsplit_30	663	101	c1_HuAgeGBsplit_691	208	181	c1_HuAgeGBsplit_66	128
22	c1_HuAgeGBsplit_64	654	102	c1_HuAgeGBsplit_338	207	182	c1_HuAgeGBsplit_303	127
23	c1_HuAgeGBsplit_17	622	103	c1_HuAgeGBsplit_408	205	183	c1_HuAgeGBsplit_40	127
24	c1_HuAgeGBsplit_124	612	104	c1_HuAgeGBsplit_1105	204	184	c1_HuAgeGBsplit_1211	126
25	c1_HuAgeGBsplit_23	602	105	c1_HuAgeGBsplit_446	204	185	c1_HuAgeGBsplit_155	126
26	c1_HuAgeGBsplit_8	582	106	c1_HuAgeGBsplit_49	202	186	c1_HuAgeGBsplit_341	126
27	c1_HuAgeGBsplit_18	571	107	c1_HuAgeGBsplit_292	201	187	c1_HuAgeGBsplit_103	125,
28	c1_HuAgeGBsplit_74	569	108	c1_HuAgeGBsplit_42	201	188	c1_HuAgeGBsplit_159	125
29	c1_HuAgeGBsplit_343	567	109	c1_HuAgeGBsplit_352	198	189	c1_HuAgeGBsplit_368	124
30	c1_HuAgeGBsplit_98	535	110	c1_HuAgeGBsplit_395	198	190	c1_HuAgeGBsplit_4ii	124
31	c1_HuAgeGBsplit_38	528	111	c1_HuAgeGBsplit_156	196	191	c1_HuAgeGBsplit_1614	123
32	c1_HuAgeGBsplit_57	524	112	c1_HuAgeGBsplit_50	194	192	c1_HuAgeGBsplit_459	122
33	c1_HuAgeGBsplit_53	519	113	c1_HuAgeGBsplit_332	192	193	c1_HuAgeGBsplit_776	122
34	c1_HuAgeGBsplit_19	517	114	c1_HuAgeGBsplit_215	190	194	c1_HuAgeGBsplit_1129	121
35	c1_HuAgeGBsplit_188	500	115	c1_HuAgeGBsplit_2103	185	195	c1_HuAgeGBsplit_830	120
36	c1_HuAgeGBsplit_360	487	116	c1_HuAgeGBsplit_2024	184	196	c1_HuAgeGBsplit_107	119
37	c1_HuAgeGBsplit_71	487	117	c1_HuAgeGBsplit_106	183	197	c1_HuAgeGBsplit_164	119
38	c1_HuAgeGBsplit_528	*484*	118	c1_HuAgeGBsplit_51	182	198	c1_HuAgeGBsplit_1032	117
39	c1_HuAgeGBsplit_29	479	119	c1_HuAgeGBsplit_160	180	199	c1_HuAgeGBsplit_178	117
40	c1_HuAgeGBsplit_103	455	120	c1_HuAgeGBsplit_79	180	200	c1_HuAgeGBsplit_764	116
41	c1_HuAgeGBsplit_14	447	121	c1_HuAgeGBsplit_1106	179	201	c1_HuAgeGBsplit_515	115
42	c1_HuAgeGBsplit_12	443	122	c1_HuAgeGBsplit_291	177	202	c1_HuAgeGBsplit_832	115
43	c1_HuAgeGBsplit_94	423	123	c1_HuAgeGBsplit_75	176	203	c1_HuAgeGBsplit_2184	114
44	c1_HuAgeGBsplit_37	421	124	c1_HuAgeGBsplit_41	175	204	c1_HuAgeGBsplit_456	114
45	c1_HuAgeGBsplit_163	412	125	c1_HuAgeGBsplit_473	170	205	c1_HuAgeGBsplit_97	114
46	c1_HuAgeGBsplit_54	405	126	c1_HuAgeGBsplit_70	170	206	c1_HuAgeGBsplit_356	123
47	c1_HuAgeGBsplit_388	389	127	c1_HuAgeGBsplit_101	167	207	c1_HuAgeGBsplit_295	111
48	c1_HuAgeGBsplit_186	383	128	c1_HuAgeGBsplit_339	167	208	c1_HuAgeGBsplit_470	111
49	c1_HuAgeGBsplit_36	353	129	c1_HuAgeGBsplit_192	166	209	c1_HuAgeGBsplit_757	111
50	c1_HuAgeGBsplit_364	347	130	c1_HuAgeGBsplit_428	166	210	c1_HuAgeGBsplit_153	110
51	c1_HuAgeGBsplit_33	345	131	c1_HuAgeGBsplit_56	164	211	c1_HuAgeGBsplit_549	110
52	c1_HuAgeGBsplit_60	340	132	c1_HuAgeGBsplit_96	163	212	c1_HuAgeGBsplit_44	110
53	c1_HuAgeGBsplit_1177	339	133	c1_HuAgeGBsplit_189	162	213	c1_HuAgeGBsplit_86	109
54	c1_HuAgeGBsplit_362	338	1.34	c1_HuAgeGBsplit_214	162	214	c1_HuAgeGBsplit_1247	108
55	c1_HuAgeGBsplit_61	335	135	c1_HuAgeGBsplit_105	160	215	c1_HuAgeGBsplit_130	108
56	c1_HuAgeGBsplit_85	334	136	c1_HuAgeGBsplit_162	160	216	c1_HuAgeGBsplit_405	108
57	c1_HuAgeGBsplit_223	323	137	c1_HuAgeGBsplit_217	159	217	c1_HuAgeGBsplit_576	108
58	c1_HuAgeGBsplit_95	321	138	c1_HuAgeGBsplit_2436	159	218	c1_HuAgeGBsplit_1132	106
59	c1_HuAgeGBsplit_501	320	139	c1_HuAgeGBsplit_372	157	219	c1_HuAgeGBsplit_1353	106
60	c1_HuAgeGBsplit_586	308	140	c1_HuAgeGBsplit_477	157	220	c1_HuAgeGBsplit_342	106
61	c1_HuAgeGBsplit_475	307	141	c1_HuAgeGBsplit_68	157	221	c1_HuAgeGBsplit_52	106
62	c1_HuAgeGBsplit_1114	304	142	c1_HuAgeGBsplit_62	156	222	c1_HuAgeGBsplit_1147	105
63	c1_HuAgeGBsplit_1293	303	143	c1_HuAgeGBsplit_151	155	223	c1_HuAgeGBsplit_125	105
64	c1_HuAgeGBsplit_32	302	144	c1_HuAgeGBsplit_622	155	224	c1_HuAgeGBsplit_228	105
65	c1_HuAgeGBsplit_73	298	145	c1_HuAgeGBsplit_1118	152	225	c1_HuAgeGBsplit_361	105
66	c1_HuAgeGBsplit_28	293	146	c1_HuAgeGBsplit_46	152	226	c1_HuAgeGBsplit_553	105
67	c1_HuAgeGBsplit_43	293	147	c1_HuAgeGBsplit_518	151	227	c1_HuAgeGBsplit_2034	102
68	c1_HuAgeGBsplit_1513	288	148	c1_HuAgeGBsplit_964	151	228	c1_HuAgeGBsplit_128	101
69	c1_HuAgeGBsplit_1047	286	149	c1_HuAgeGBsplit_47	150	229	c1_HuAgeGBsplit_358	101
70	c1_HuAgeGBsplit_398	280	150	c1_HuAgeGBsplit_63	150	230	c1_HuAgeGBsplit_605	101
71	c1_HuAgeGBsplit_89	273	151	c1_HuAgeGBsplit_690	150	231	c1_HuAgeGBsplit_82	101
72	c1_HuAgeGBsplit_628	271	152	c1_HuAgeGBsplit_99	150	232	c1_HuAgeGBsplit_93	101
73	c1_HuAgeGBsplit_67	269	153	c1_HuAgeGBsplit_254	148	233	c1_HuAgeGBsplit_1081	100
74	c1_HuAgeGBsplit_174	268	154	c1_HuAgeGBsplit_158	147	234	c1_HuAgeGBsplit_181	100
75	c1_HuAgeGBsplit_35	267	155	c1_HuAgeGBsplit_277	147	235	c1_HuAgeGBsplit_1895	100
76	c1_HuAgeGBsplit_45	260	156	c1_HuAgeGBsplit_451	145			
77	c1_HuAgeGBsplit_31	259	157	c1_HuAgeGBsplit_384	144			
78	c1_HuAgeGBsplit_77	259	158	c1_HuAgeGBsplit_675	144			
79	c1_HuAgeGBsplit_39	257	159	c1_HuAgeGBsplit_78	144			
80	c1_HuAgeGBsplit_34	256	160	c1_HuAgeGBsplit_253	143			

**Figure 2:**
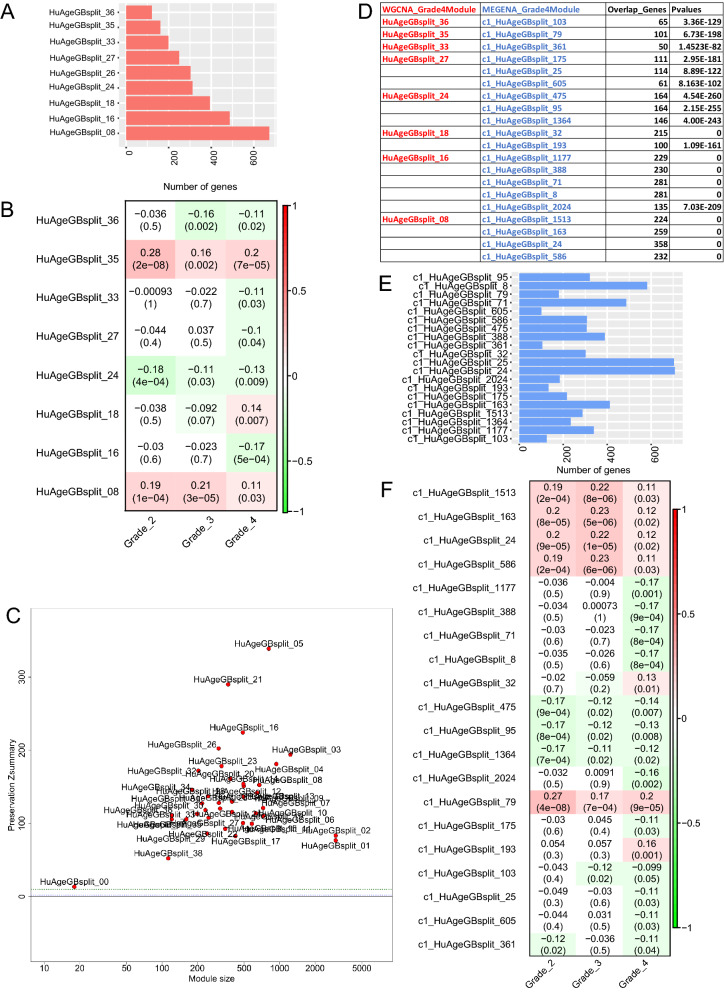
Identification of WGCNA and MEGENA Glioblastoma modules. (**A**) Bar-plot showing number of genes in Glioblastoma associated WGCNA modules. (**B**) Spearman correlation coefficient and p-values for significant tumor Grade 4 or Glioblastoma associated WGCNA modules (**C**) Preservation analysis between all WGCNA modules and all MEGENA modules using WGCNA module labels. (**D**) Table showing MEGENA modules that significantly overlapped with WGCNA Glioblastoma associated modules. (**E**) Bar-plot showing number of genes in Glioblastoma associated MEGENA modules that significantly overlap with Glioblastoma associated WGCNA modules. (**F**) Spearman correlation coefficient and p-values for significant tumor Grade 4 or Glioblastoma associated MEGENA modules that also significantly overlap with tumor Grade 4 or Glioblastoma associated WGCNA modules.

To identify GBM associated modules, a module eigengene was used to represent overall expression pattern of each module produced by WGCNA and MEGENA. Spearman correlations were calculated for GBM and other clinical traits, such as batch, age, and gender. Eight modules in WGCNA, with module size ranging from 673 genes in HuAgeGBsplit_08 to 121 genes in Hu_AgeGBsplit_36, were found to be significantly associated with GBM (Fig. 2A,B). All WGCNA modules, including GBM associated modules, were preserved with MEGENA modules (Fig. 2C). Comparison of GBM specific WGCNA modules with MEGENA modules showed a significant overlap of all WGCNA GBM modules with MEGENA modules (Fig. 2D). All 20 MEGENA modules that significantly overlapped with GBM WGCNA modules, with module size ranging from 101 genes in c1_HuAgeGBsplit_605 to 708 genes in c1_HuAgeGBsplit_24, were also significantly associated with GBM (Fig. 2E,F). Thus, WGCNA and MEGENA complemented each other and helped identify GBM specific modules in largely preserved glioma WGCNA and MEGENA gene networks.

### Differential gene expression analysis reveals proliferative and quiescent stem cell marker genes

**Figure 3:**
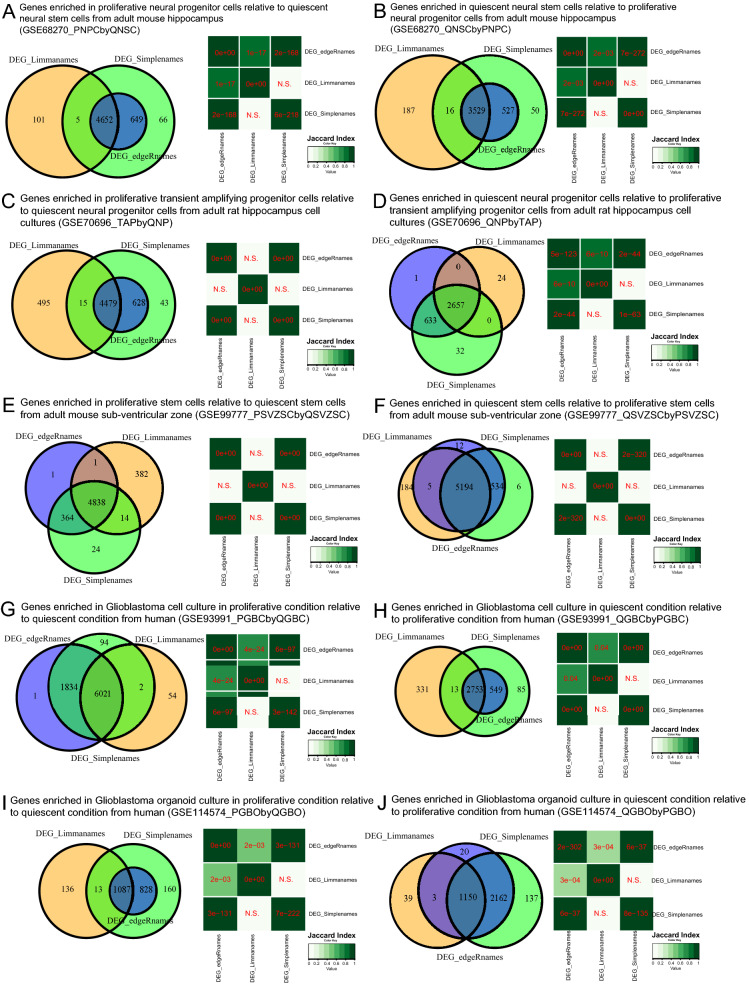
(**A–J**) Venn Diagram showing number of overlapping genes and heat-map showing significance of overlap for different differential gene expression calculation methods. Upregulated differentially expressed genes (DEGs) for comparisons between GSE68270_PNPCbyQNSC (**A**), GSE68270_QNSCbyPNPC (**B**), GSE70696_TAPbyQNP (**C**), GSE70696_QNbyTAP (**D**), GSE99777_PSVZSCbyQSVZSC (**E**), GSE99777_QSVZSCbyPSVZSC (**F**), GSE93991_PGBCbyQGBC (**G**), GSE93991_QGBCbyPGBC (**H**), GSE114574_PGBObyQGBO (**I**) and GSE114574_QGBObyPGBO (**J**) as determined by Limma, edgeR and simple comparison of means methods are shown.

To identify genes specific to quiescent and proliferative states of stem cells, differential gene expression analysis was performed on different stem cell datasets as described under methods. In mouse adult hippocampal stem cell dataset, 5,306 (3,959 human gene symbols) and 4,072 (3,188 human gene symbols) DEGs with a fold change of 1.25 (p-value < 0.05) were identified in proliferation (GSE68270_PNPCbyQNSC) and quiescence (GSE68270_PNPCbyQNSC), respectively (Fig. 3A,B). In rat adult hippocampal stem cell dataset, 5,122 (4,362 human gene symbols) and 3,290 (2,792 human gene symbols) DEGs with a fold change of 1.25 (p-value < 0.05) were identified in proliferation (GSE70696_TAPbyQNP) and quiescence (GSE70696_QNPbyTAP), respectively (Fig. 3C,D). In mouse adult subventricular zone stem cell dataset, 5,216 (4,235 human gene symbols) and 5,733 (4,658 human gene symbols) DEGs with a fold change of 1.25 (p-value < 0.05) were identified in proliferation (GSE99777_PSVZSCbyQSVZSC) and quiescence (GSE99777_QSVZSCbyPSVZSC), respectively (Fig. 3E,F). In human GBM cell culture dataset, 7,857 and 3,363 (human gene symbols) DEGs with a fold change of 1.25 (p-value < 0.05) were identified in proliferation (GSE93991_PGBCbyQGBC) and quiescence (GSE93991_QGBCbyPGBC), respectively (Fig. 3G,H). In human GBM organoid culture dataset, 1928 and 3,315 (human gene symbols) DEGs with a fold change of 1.25 (p-value < 0.05) were identified in proliferation (GSE114574_PGBObyQGBO) and quiescence (GSE114574_QGBObyPGBO), respectively (Fig. 3I,J). Stem cell marker genes common between all normal stem cells and GBM culture datasets (GSE68270, GSE70696, GSE99777, GSE93991 and GSE114574), consisted of 7 proliferation genes or DEGs (ACYP1, AKAP12, LRP11, MYSM1, SLC20A1, TERT, TSPAN13) and 5 quiescence genes or DEGs (ETHE1, FZD9, NINJ1, P2RX4, PTP4A3) (Tables 3, 4). Overlapping stem cell marker genes obtained from normal stem cell datasets (GSE68270, GSE70696 and GSE99777), with GBM culture datasets (GSE93991 and GSE114574), showed an overlap of 4,176 proliferation genes (45.87% of GBM proliferation DEGs) and only 1598 quiescence genes (25.97% of GBM quiescence DEGs (Supplementary Tables S1A,B).

**Table 3:** Common stem cell markers of proliferation.

Gene_Symbol	Common to DEGs listed below from normal stem cells and Glioblastoma in proliferative conditions
ACYP1	GSE68270_PNPCbyQNSC, GSE70696_TAPbyQNP, GSE99777_PSVZSCbyQSVZSC, GSE93991_PGBCbyQGBC, GSE114574_PGBObyQGBO
AKAP12	GSE68270_PNPCbyQNSC, GSE70696_TAPbyQNP, GSE99777_PSVZSCbyQSVZSC, GSE93991_PGBCbyQGBC, GSE114574_PGBObyQGBO
LRP11	GSE68270_PNPCbyQNSC, GSE70696_TAPbyQNP, GSE99777_PSVZSCbyQSVZSC, GSE93991_PGBCbyQGBC, GSE114574_PGBObyQGBO
MYSM1	GSE68270_PNPCbyQNSC, GSE70696_TAPbyQNP, GSE99777_PSVZSCbyQSVZSC, GSE93991_PGBCbyQGBC, GSE114574_PGBObyQGBO
SLC20A1	GSE68270_PNPCbyQNSC, GSE70696_TAPbyQNP, GSE99777_PSVZSCbyQSVZSC, GSE93991_PGBCbyQGBC, GSE114574_PGBObyQGBO
TERT	GSE68270_PNPCbyQNSC, GSE70696_TAPbyQNP, GSE99777_PSVZSCbyQSVZSC, GSE93991_PGBCbyQGBC, GSE114574_PGBObyQGBO
TSPAN13	GSE68270_PNPCbyQNSC, GSE70696_TAPbyQNP, GSE99777_PSVZSCbyQSVZSC, GSE93991_PGBCbyQGBC, GSE114574_PGBObyQGBO

**Table 4:** Common stem cell markers of quiescence.

Gene_Symbol	Common to DEGs listed below from normal stem cells and Glioblastoma in quiescent conditions
ETHE1	GSE68270_QNSCbyPNPC, GSE70696_QNPbyTAP, GSE9777_QSVZSCbyPSVZSC, GSE93991_QGBCbyPGBC, GSE114574_QGBObyPGBO
FZD9	GSE68270_QNSCbyPNPC, GSE70696_QNPbyTAP, GSE9777_QSVZSCbyPSVZSC, GSE93991_QGBCbyPGBC, GSE114574_QGBObyPGBO
NINJ1	GSE68270_QNSCbyPNPC, GSE70696_QNPbyTAP, GSE9777_QSVZSCbyPSVZSC, GSE93991_QGBCbyPGBC, GSE114574_QGBObyPGBO
P2RX4	GSE68270_QNSCbyPNPC, GSE70696_QNPbyTAP, GSE9777_QSVZSCbyPSVZSC, GSE93991_QGBCbyPGBC, GSE114574_QGBObyPGBO
PTP4A3	GSE68270_QNSCbyPNPC, GSE70696_QNPbyTAP, GSE9777_QSVZSCbyPSVZSC, GSE93991_QGBCbyPGBC, GSE114574_QGBObyPGBO

### Proliferative and quiescent stem cell marker genes underly GBM modules

**Figure 4:**
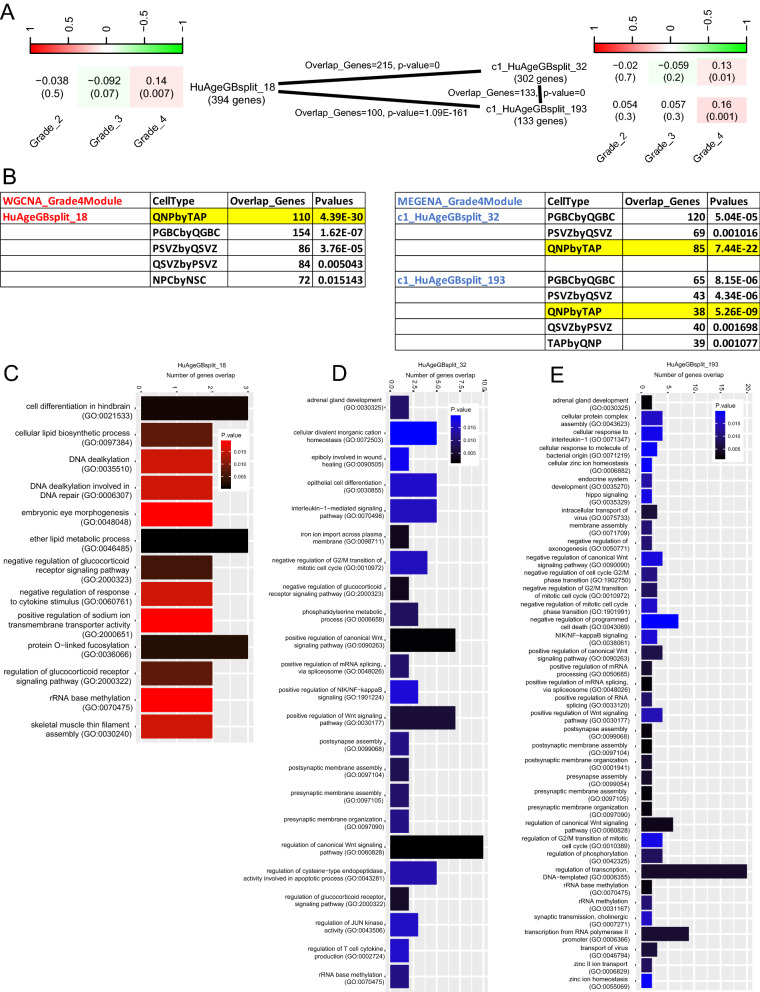
Stem cell genes enriched WGCNA and MEGENA Glioblastoma modules. (**A**) Number of genes overlapping between Glioblastoma modules, HuAgeGBsplit_18 WGCNA and its equivalent c1_HuAgeGBsplit_32, and HuAgeGBsplit_193 MEGENA modules. HuAgeGBsplit_193 is a subset of HuAgeGBsplit_32 MEGENA module. (**B**) Table showing most significant overlap of rat quiescent hippocampal stem cell genes (QNPbyTAP) in Glioblastoma associated HuAgeGBsplit_18 WGCNA and c1_HuAgeGBaplit_32/193 MEGENA modules. (**C**, **D**, **E**) Gene Ontology (GO) Biological Process analysis of quiescent stem cell (QNPbyTAP) signature enriched HuAgeGBsplit_18 (**C**), c1_HuAgeGBsplit_32 (**D**) and c1_HuAgeGBsplit_193 (**E**).

Hypergeometric test showed that HuAgeGBsplit_18 WGCNA GBM module and its equivalent c1_HuAgeGBsplit_193/32 MEGENA GBM modules, were the only GBM modules significantly enriched with proliferative and quiescent stem cell marker genes (Fig. 4A,B). Comparison of genes in c1_HuAgeGBsplit_193 and c1_HuAgeGBsplit_32 MEGENA modules, revealed that all genes in c1_HuAgeGBsplit_193 were also present in c1_HuAgeGBsplit_32 (Fig. 4A). Though proliferative and quiescent stem cell markers from different stem cell datasets (GSE68270, GSE70696, GSE99777, GSE93991 and GSE114574) were enriched in GBM modules (HuAgeGBsplit_18 WGCNA and c1_HuAgeGBsplit_193/32 MEGENA), quiescent adult hippocampal rat stem cell marker genes (GSE70696_QNPbyTAP) were most significantly enriched (Fig. 4B). However, no significant enrichment of SWIM 336 Glioblastoma gene list was found in HuAgeGBsplit_18 WGCNA module (p-value 0.411, overlap of 9 genes) and its equivalent MEGENA modules, c1_HuAgeGBsplit_32/193 MEGENA modules (p-value 1, overlap of 6 genes in c1_HuAgeGBsplit_32 and p-value 1, overlap of 2 genes in c1_HuAgeGBsplit_193).

### Gene ontology (GO) annotation of GBM modules enriched with quiescent stem cell marker genes

To determine biological processes underlying GBM modules (HuAgeGBsplit_18 WGCNA and c1_HuAgeGBsplit_32/193 MEGENA modules) enriched with quiescent stem cell marker genes (GSE70696_QNPbyTAP), biological process gene ontology (GO) analysis was done. Lipid metabolic processes, “ether lipid metabolic process (GO:0046485)” and “cellular lipid biosynthetic process (GO:0097384)”, signaling pathways, “regulation of glucocorticoid receptor signaling pathway (GO:2000322)” and “negative regulation of response to cytokine stimulus (GO:0060761)”, and biomolecule modification processes, "protein O-linked fucosylation (GO:0036066)", "DNA dealkylation (GO:0035510)" and "rRNA base methylation (GO:0070475)", were top hits in HuAgeGBsplit_18 (Fig. 4C). Lipid metabolic processes, “phosphatidylserine metabolic process (GO:0006658)”, signaling pathways, “regulation of canonical Wnt signaling pathway (GO:0060828)” and “regulation of glucocorticoid receptor signaling pathway (GO:2000322)”, and biomolecule modification processes, “rRNA base methylation (GO:0070475)” and “positive regulation of mRNA splicing, via spliceosome (GO:0048026)”, were top hits in c1_HuAgeGBsplit_32/193 MEGENA modules (Fig. 4D,E). Most biological process GO categories showed comparable profiles for WGCNA and MEGENA GBM modules (HuAgeGBsplit_18 WGCNA and c1_HuAgeGBsplit_32/193 MEGENA modules) enriched with quiescent stem cell marker genes (GSE70696_QNPbyTAP). This suggests that networks identified by WGCNA and MEGENA network analysis are consistent and biologically robust.

### Logistic regression model built with quiescent stem cell marker genes in GBM modules

**Figure 5:**
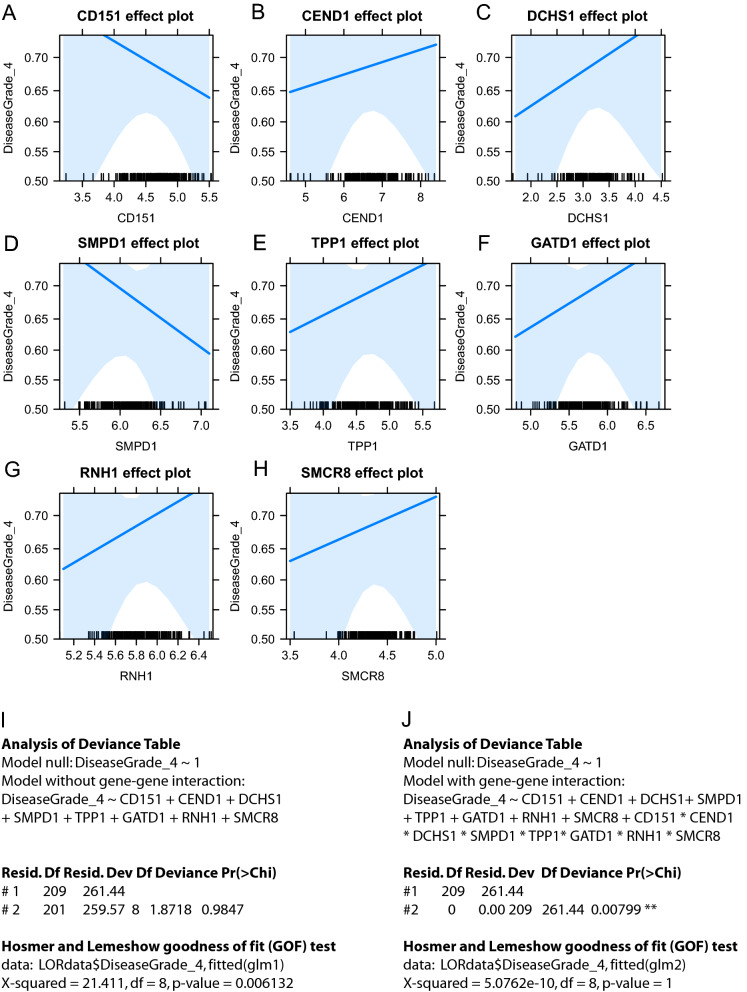
Logistic regression model for prediction of Glioblastoma using QNPbyTAP genes that are atleast 20-fold-upregulated in QNP relative to TAP and are significantly enriched in in Glioblastoma HuAgeGBsplit_18 WGCNA module. Effects plot showing probability of Glioblastoma (DiseaseGrade_4) with change in expression of CD151 (**A**), CEND1 (**B**), DCHS1 (**C**), SMPD1 (**D**), TPP1 (**E**), GATD1 (**F**), RNH1 (**G**) and SMCR8 (**H**). Chi-square test and Hosmer and Lemeshow goodness of fit (GOF) test for logistic regression model without gene–gene interaction (**I**) and with gene–gene interaction (**J**).

A logistic regression model was built with select quiescent stem cell marker genes (GSE70696_QNPbyTAP) to diagnostic between control and GBM samples. From a total of 110 genes from GSE70696_QNPbyTAP enriched in GBM WGCNA module HuAgeGBsplit_18, genes that were atleast 40-fold upregulated in QNP relative to TAP were selected (CD151, CEND1, DCHS1, SMPD1, TPP1, GATD1, RNH1 and SMCR8) for logistic regression. Effects plots showed that probability of GBM relative to control increases with increased expression of CEND1, DCHS1, TPP1, GATD1, RNH1 and SMCR8 (Fig. 5B,C,E–H) and decreases with increased expression of CD151 and SMPD1 (Fig. 5A,D). Logistic regression model with these 8 genes without gene–gene interaction term was not significant (Chi-square p-value = 0.9847), while with gene–gene interactions the model was a significant (Chi-square p-value = 0.00799) predictor for GBM (Fig. 5I,J).

Hosmer–Lemeshow GOF test on without gene–gene interaction logistic regression model showed a large difference between observed and expected probabilities, and there was significant evidence of poor fit (p-value = 0.006, less than 0.05) (Fig. 5I, Table 5). Therefore, in logistic regression model without gene–gene interaction, Ho is rejected and Ha is accepted, and the model is rejected for being a poor fit for the data. Hosmer–Lemeshow GOF test on with gene–gene interaction logistic regression model showed a small difference between observed and expected probabilities, and there was no significant evidence of poor fit (p-value = 1, greater than 0.05) (Fig. 5J, Table 6). Therefore, in logistic regression model with gene–gene interaction, Ho is accepted and the model is accepted as a good fit for the data.

**Table 5:** Hosmer and Lemeshow goodness of fit (GOF) test for model without gene–gene interaction shows significant difference between observed and expected probabilities (p-value = 0.006132, < 0.05).

Observed probability value y0	Observed probability value y1	Expected probability value yhat0	Expected probability value yhat1	Difference (y0-yhat0)	Difference (y1-yhat1)
4	17	8.256231	12.74377	− 4.25623	4.256231
4	17	7.515885	13.48411	− 3.51589	3.515885
11	10	7.174084	13.82592	3.825916	− 3.82592
12	9	6.914975	14.08503	5.085025	− 5.08503
9	12	6.714101	14.2859	2.285899	− 2.2859
6	15	6.50056	14.49944	− 0.50056	0.50056
6	15	6.24459	14.75541	− 0.24459	0.24459
8	13	5.974346	15.02565	2.025654	− 2.02565
5	16	5.650239	15.34976	− 0.65024	0.650239
1	20	5.054989	15.94501	− 4.05499	4.054989

Model without gene–gene interaction: DiseaseGrade_4 ~ CD151 + CEND1 + DCHS1 + SMPD1 + TPP1 + GATD1 + RNH1 + SMCR8.

**Table 6:** Hosmer and Lemeshow goodness of fit (GOF) test for model with gene–gene interaction shows no significant difference (almost zero difference) between observed and expected probabilities (p-value = 1, > 0.05).

Observed probability value y0	Observed probability value y1	Expected probability value yhat0	Expected probability value yhat1	Difference (y0-yhat0)	Difference (y1-yhat1)
21	0	21	6.09E−11	6.09E−11	− 6.09E−11
21	0	21	6.09E−11	6.09E−11	− 6.09E−11
21	0	21	6.09E−11	6.09E−11	− 6.09E−11
3	32	3	32	− 8.41E−11	8.41E−11
0	43	1.25E−10	43	− 1.25E−10	1.25E−10
0	52	1.51E−10	52	− 1.51E−10	1.51E−10
0	17	4.93E−11	17	− 4.93E−11	4.93E−11

Model with gene–gene interaction: DiseaseGrade_4 ~ CD151 + CEND1 + DCHS1 + SMPD1 + TPP1 + GATD1 + RNH1 + SMCR8 + CD151 * CEND1 * DCHS1 * SMPD1 * TPP1 * GATD1 * RNH1 * SMCR8.

## Discussion

Tumors are commonly treated by surgical removal, chemotherapy, radiotherapy and immunotherapy^57^. Safe surgical removal of glioma is challenging due to its critical location in brain. Additionally, high grade glioma or GBM is highly resistant to chemotherapy and radiotherapy. Immunotherapy treatments are FDA approved for certain blood cancers, but for solid tumors such as GBM immunotherapy ineffective due to incomplete infiltration of immunotherapeutic agent and immune suppression by tumor microenvironment^58,59^. Diagnosis is the first step in development of an effective treatment plan for any disease, including glioma. GBM is the most aggressive form of glioma that advances quickly giving healthcare providers limited time for diagnosis and treatment^60^. Therefore, to gain deeper understanding of glioma, especially GBM, with hope to develop early accurate diagnosis tools and novel therapies, molecular profiling and network medicine have emerged as research forerunners.

Molecular profile or gene based classification and diagnosis help physicians plan treatment and predict clinical outcome. For example, IDH1 gene mutation is highly correlated with glioma survival and is therefore used for glioma classification^61^. IDH1 mutation is lowest in grade 1 glioma that correlates with slow tumor growth and good survival^4^. On the other hand, IDH1 mutation is highest in grade 4 glioma or GBM that correlates with fast tumor growth and poor survival^4^. Improvement in technology, reduced cost of high-throughput sequencing, extensive collaborations and data sharing have made a plethora of glioma molecular profiling datasets such as RNA-seq available to research community^18,20^. With the explosion of molecular profiling genomics big data, research focus has now shifted from big data mining to big data analysis to prioritize a set of genes with diagnostic value that would eliminate need to profile all 23 K protein coding genes from glioma samples. However, prioritization of a subset of genes for glioma diagnosis and classification has been challenging due to high cellular heterogeneity across and within tumor samples of glioma^62^. Stem cell-like cells in glioma are thought to be responsible for tumor initiation, progression and recurrence^63^. Chemotherapy and radiotherapy kill proliferative stem cells, but are unable to kill quiescent stem cells in the tumor. Quiescent stem cells left in the tumor at end of treatment enter a proliferative state and reconstitute tumor, which leads to tumor recurrence^64^. Therefore, here the goal was to identify distinct sets of proliferative and quiescent stem cell marker genes in GBM that can be used for diagnosis and can serve as potential drug targets.

A meta-analysis was performed on publicly available high-throughput gene expression datasets from human glioma samples, control human brains, normal stem cells and GBM cells in quiescent and proliferative states^18,26–34^. DEGs specific to stem cell states were identified from normal stem cell and Glioblastoma cell culture datasets in quiescent and proliferative states. Interestingly, only 45.87% and 25.97% of genes from GBM cell cultures in proliferative and quiescent states were common with normal stem cells in proliferative and quiescent states, respectively (Supplementary Table S1 A,B). This suggests that cancer stem cells, especially those in quiescent state are distinctly different from normal stem cells.

Network analysis facilitates grouping of genes with highly correlated gene expression patterns into modules. It is assumed that modules corelate with distinct biological and cellular states, such as diseases and cell types, respectively. Presently, network analysis identified 9 WGCNA modules and 20 MEGENA modules that were highly correlated with GBM (Fig. 2B,F). One of these WGCNA modules, HuAgeGBsplit_18 WGCNA module (equivalent c1_HuAgeGBsplit_32/193 MEGENA modules) was also significantly enriched with adult hippocampal rodent quiescent stem cell genes (GSE70696_QNPbyTAP) (Fig. 4B). Interestingly, though this quiescent stem cell marker enriched HuAgeGBsplit_18 WGCNA module (equivalent c1_HuAgeGBsplit_32/193 MEGENA modules) had a significant correlation with GBM (p-value 0.007) it had a small correlation value of 0.14 with GBM (Fig. 2B). Possible reasons for this small but significant correlation are discussed here: (A) The result is consistent between WGCNA and MEGENA, two completely different network analysis algorithms. This supports that the results are biologically robust and not a computational artifact that would alter based on alterations in default algorithm settings. (B) SVA + LM normalization was used in this study to retain effects of glioma on gene expression and remove effects of all other covariants. It is possible that effects of covariants such as batch effects are not completely removed by this normalization, which is confounding glioma gene expression effects. (C) It is possible there are other covariants that significantly effect gene expression, such as patients’ comorbidities. However, as this information was not available, it could not be included in SVA + LM normalization and therefore glioma effects could not be effectively retained. (D) Controls used in this study comprise of RNA-seq datasets from different parts of the brain derived from humans other than the patients themselves. Ideally control tissue should be derived from the same patient who has the glioma, but presently such patient matched controls were not available for analysis. Lack of patient matched controls is a common challenge in the field of glioma and human disease research.

Quiescent stem cells exist in non-proliferative G0 cell cycle phase, but retain ability to reversibly enter cell cycle in response to stimuli. Depletion of surrounding proliferative stem cells and differentiated cells stimulate quiescent stem cells, which are multipotent and have self-renewal potential, to enter cell cycle and replenish the tissue^65^. Quiescent stem cell properties of rodent hippocampal stem cells have been extensively experimentally characterized^33,66^. Though proliferative stem cell marker genes of GBM origin (PGBCbyQGBC) were significantly enriched in HuAgeGBsplit_18 WGCNA GBM module (equivalent c1_HuAgeGBsplit_32/193 MEGENA GBM modules) with p-value 1.62E-07, it was normal quiescent stem cell marker genes from rodent hippocampal stem cells (GSE70696_QNPbyTAP) that were most significantly enriched with p-value 4.39E-20 (Fig. 4B). Normal stem cells transition from quiescent state to proliferative state and further to differentiated state. Recently, a set of 336 genes were identified in GBM with SWIM network analysis method, which are potentially involved in transition from stem-like state to differentiated state^52,53^. Interestingly, no significant enrichment (p-value 0.411, overlap of 9 genes) of SWIM 336 Glioblastoma gene list was found in HuAgeGBsplit_18 GBM WGCNA module (equivalent c1_HuAgeGBsplit_32/193 MEGENA GBM modules). This supports that quiescent stem cell marker genes (GSE70696_QNPbyTAP) enriched in HuAgeGBsplit_18 WGCNA GBM module (equivalent c1_HuAgeGBsplit_32/193 MEGENA GBM modules) represent an undifferentiated quiescent stem cell state distinct from differentiating stem cells.

Gene Ontology (GO) analysis of GSE70696_QNPbyTAP enriched HuAgeGBsplit_18 WGCNA GBM module (equivalent c1_HuAgeGBsplit_32/193 MEGENA GBM modules) revealed enrichment of biomolecule synthesis GO terms, such as lipid metabolism, DNA modification, protein post-translational modification, ribosome RNA processing, cell cycle GO terms (G2/M transition, mitotic cell cycle) and signaling pathways such as Wnt signaling (Fig. 4C–E). This is consistent with cell cycle and ribosome biogenesis GO terms, previously reported in the rodent hippocampal stem cell dataset from which GSE70696_QNPbyTAP signature genes were identified^33^. As quiescent stem cells can replenish tumor after proliferative stem cells are killed by chemotherapy and radiotherapy, a combinatorial therapy that targets both proliferative and quiescent stem cells could be more effective in GBM treatment. Quiescent stem cell marker genes (GSE70696_QNPbyTAP) enriched in HuAgeGBsplit_18 WGCNA GBM module (equivalent c1_HuAgeGBsplit_32/193 MEGENA GBM modules) reported here, could serve as potential GBM quiescent stem cell drug targets. Small molecule DYRK1B inhibitors were recently shown to target quiescent stem cells and potentiate treatment benefits of chemotherapy^67^. This supports development of treatment strategies to target both proliferative and quiescent stem cells in GBM.

Gene expression of eight genes (CD151, CEND1, DCHS1, SMPD1, TPP1, GATD1, RNH1 and SMCR8) from GSE70696_QNPbyTAP enriched in HuAgeGBsplit_18 WGCNA GBM module (equivalent c1_HuAgeGBsplit_32/193 MEGENA GBM modules), were sufficient to build a logistic regression diagnostic model that could distinguish between GBM and control samples (Fig. 5J). Four of the eight genes (CD151, CEND1, SMPD1 and RNH1) used in the model have previously been reported to be important in GBM, other types of cancer or in development^68–71^. CD151 is a member of tetraspanins scaffolding protein family that is involved in cell–cell adhesion, integrin interaction, cell signaling, cancer progression and metastasis^72^. In GBM, CD151 associates with α3β1 integrin to potentiate EGFR signaling, drive cancer cell motility and tumor aggressiveness^71^. CEND1 or BM88 protein acts as a cell-cycle inhibitor to negatively regulated proliferation and promote differentiation in spinal cord development^70^. In cell membrane lipid bilayer, apoptosis and cellular growth is regulated by balance between sphingosine-1-phosphate and ceramide molecules^73^. SMPD1 gene that regulates ‘ceramide sphingosine-1-phosphate rheostat’ drives tumor growth and immune escape in non-small cell lung cancer^69^. RNH1 repairs DNA in response to DNA damage and is a known diagnostic and prognostic marker of glioma^68^. Taken together, this provides support for biological relevance of the eight genes (CD151, CEND1, DCHS1, SMPD1, TPP1, GATD1, RNH1 and SMCR8) used here to build a logistic regression diagnostic model for GBM. A molecular screening kit could be developed with these eight genes for faster and accurate screening of GBM. However, as results of present study were obtained by using computational meta-analysis alone, further experimental validation is required. Overall, this study deconvolutes highly heterogeneous glioma molecular profiles and provides a new perspective to diagnose and develop therapeutic strategies using a small number of quiescent stem cell markers in GBM.

## Supplementary information

Supplementary Table 1A

Supplementary Table 1B

## Data Availability

Glioblastoma RNA-seq datasets, SRP027383 and SRP091303, were obtained from NCBI SRA. Other RNA-seq datasets for control brains (GSE67333, GSE64810, GSE100297 and GSE53697) and stem cells (GSE68270, GSE70696, GSE99777, GSE93991 and GSE114574), were obtained from NCBI GEO. The author is most grateful to NCBI SRA and NCBI GEO for storing and making these datasets open access. The author also appreciates the organizations and investigators who generously submitted these datasets for sharing to NCBI GEO and NCBI SRA.
